# Global Actions for Managing Cactus Invasions

**DOI:** 10.3390/plants8100421

**Published:** 2019-10-16

**Authors:** Ana Novoa, Giuseppe Brundu, Michael D. Day, Vicente Deltoro, Franz Essl, Llewellyn C. Foxcroft, Guillaume Fried, Haylee Kaplan, Sabrina Kumschick, Sandy Lloyd, Elizabete Marchante, Hélia Marchante, Iain D. Paterson, Petr Pyšek, David M. Richardson, Arne Witt, Helmuth G. Zimmermann, John R. U. Wilson

**Affiliations:** 1 Centre for Invasion Biology, Department of Botany and Zoology, Stellenbosch University, Private Bag X1, Matieland 7602, South Africa; franz.essl@univie.ac.at (F.E.); llewellyn.foxcroft@sanparks.org (L.C.F.); sabrina.kumschick@gmail.com (S.K.); petr.pysek@ibot.cas.cz (P.P.); rich@sun.ac.za (D.M.R.); jrwilson@sun.ac.za (J.R.U.W.); 2 South African National Biodiversity Institute, Kirstenbosch Research Centre, Private Bag X7, Claremont 7735, South Africa; haylee.kaplan@gmail.com; 3 Institute of Botany, Department of Invasion Ecology, The Czech Academy of Sciences, CZ-252 43 Průhonice, Czech Republic; 4 Department of Agriculture, University of Sassari, 07100 Sassari, Italy; gbrundu@tin.it; 5 Queensland Department of Agriculture and Fisheries, GPO Box 267, Brisbane Qld 4001, Queensland, Australia; Michael.Day@daf.qld.gov.au; 6 VAERSA-Generalitat Valenciana, E-46011 Valencia, Spain; vdeltoro@gmail.com; 7 Division of Conservation Biology, Vegetation and Landscape Ecology, Department of Botany and Biodiversity Research, University of Vienna, 1030 Vienna, Austria; 8 Conservation Services, South African National Parks, Skukuza 1350, South Africa; 9 Anses, Laboratoire de la Santé des Végétaux, Unité Entomologie et Plantes invasives, 34988 Montferrier-sur-Lez Cedex, France; guillaume.fried@anses.fr; 10 Department of Primary Industries and Regional Development, Baron-Hay Court, South Perth 6151, Australia; 1weeds@live.com; 11 Centre for Functional Ecology, Department of Life Sciences, University of Coimbra, 3000-456 Coimbra, Portugal; elizabete.marchante@gmail.com (E.M.); hmarchante@gmail.com (H.M.); 12 Instituto Politécnico de Coimbra, Escola Superior Agrária de Coimbra, 3045-601 Coimbra, Portugal; 13 Centre for Biological Control, Department of Zoology and Entomology, PO Box 94, Rhodes University, Grahamstown 6139, South Africa; i.paterson@ru.ac.za; 14 Department of Ecology, Faculty of Science, Charles University, Viničná 7, CZ-128 44 Prague, Czech Republic; 15 CABI Africa, Nairobi 633-00621, Kenya; a.witt@cabi.org; 16 Helmuth Zimmermann & Associates, Pretoria 0043, South Africa; zimmermannhelmuth@gmail.com

**Keywords:** biological control, Cactaceae, early detection and eradication, impacts, prevention, public awareness, public engagement

## Abstract

The family Cactaceae Juss. contains some of the most widespread and damaging invasive alien plant species in the world, with Australia (39 species), South Africa (35) and Spain (24) being the main hotspots of invasion. The Global Cactus Working Group (IOBC GCWG) was launched in 2015 to improve international collaboration and identify key actions that can be taken to limit the impacts caused by cactus invasions worldwide. Based on the results of an on-line survey, information collated from a review of the scientific and grey literature, expertise of the authors, and because invasiveness appears to vary predictably across the family, we (the IOBC GCWG): (1) recommend that invasive and potentially invasive cacti are regulated, and to assist with this, propose five risk categories; (2) recommend that cactus invasions are treated physically or chemically before they become widespread; (3) advocate the use of biological control to manage widespread invasive species; and (4) encourage the development of public awareness and engagement initiatives to integrate all available knowledge and perspectives in the development and implementation of management actions, and address conflicts of interest, especially with the agricultural and ornamental sectors. Implementing these recommendations will require global co-operation. The IOBC GCWG aims to assist with this process through the dissemination of information and experience.

## 1. Introduction and Methods

Humans have been introducing species to areas outside their native ranges for centuries [1,2]. Although only a fraction of the introduced taxa become invasive, invasive species can cause significant negative environmental and socioeconomic impacts in invaded areas [3,4,5,6,7,8]. Management actions are therefore underway in many parts of the world [9] to achieve one or more of three main goals: prevention (to regulate potential invaders through national and/or international policies and control their introduction at ports of entry), eradication (to find and completely remove invasive species from a region), and impact reduction (to manage invasions to contain their spread and reduce their impacts).

The funding and capacity required to manage all invasive alien species usually exceeds available resources. A useful approach for prioritising the allocation of resources is to develop management actions for groups of species with similar management requirements rather than developing and implementing separate strategies for each species [10,11]. If invasive species have similar characteristics and impacts, share common stakeholders, invade similar environments and require similar management responses, grouping them for management purposes (termed “invasion syndromes” [12]) could simplify the decision-making process. Sharing lessons, approaches, and techniques can thereby reduce management costs. Collaboration between countries can also reduce costs, since lessons gained from the successes and failures of management in one country can guide managers in others [13,14].

Here, we focus on the plant family Cactaceae Juss., commonly referred to as cacti, which has a number of widespread invasive alien species in different parts of the world [15]. We review and unify knowledge on the actions implemented worldwide to manage invasive alien cacti, thereby contributing towards the development of management strategies to mitigate the negative impacts of cactus invasions. The family Cactaceae contains 1919 succulent plant species native to the American continent [16], although the native range of *Rhipsalis baccifera* (Mill) Stearn is still unclear [17]. More than 200 cactus species have been introduced outside their native range for human consumption, animal fodder, and for medicinal and ornamental purposes [18]. While many species do not become problematic, 57 cactus species are currently listed as invasive around the world, with Australia (39 species), South Africa (35) and Spain (24) representing the hotspots of cacti invasion [15]. In the invaded areas, invasive cacti cause a range of negative impacts. For example, on biodiversity, national economies, and human health [19,20]. Moreover, with 97 naturalised species reported globally, Cactaceae rank among the top 30 families with the most naturalised aliens [2].

Cactus invasions were amongst the first plant invasions to be recognised and regulated. Cacti were the first plants targeted for classical biological control, with management efforts dating back to the 1800s [21]. Some of these early interventions were extremely successful, such as the biological control programmes against *Opuntia stricta* (Haw.) Haw. in Australia and South Africa [22,23]. Management of invasive cacti has continued, stimulating increasing efforts to improve collaboration. For example, in Australia in 2009, representatives from various government biosecurity agencies, the pest management community, the Rangelands Alliance, the scientific community, and the South Australian State Opuntia Taskforce formed the Australian Invasive Cacti Network (AICN; http://www.aicn.org.au). The main aims of the national network are to raise awareness of the impacts of invasive cacti in the country and to provide a forum for exchanging information on the taxonomy, biology and management of invasive cacti. Nowadays, the AICN consists of more than 100 members from all mainland states of Australia. Similarly, in South Africa, a national working group (the South African Cactus Working Group; SACWG) was established in 2013 [24]. The SACWG consists of representatives from all relevant organisations in South Africa involved in research, policy, and management of cactus invasions. The main aims of the SACWG are to inform ongoing research and interventions and, similar to the AICN, to exchange ideas and current knowledge among experts on cactus invasions.

To build on these national initiatives, the International Organization of Biological Control (IOBC) launched the Global Cactus Working Group (IOBC GCWG) in 2015. The main aims of the IOBC GCWG are to share, design, discuss, and promote best management practices of cacti in their introduced ranges (https://www.iobc-global.org/global_wg_cactus.html).

A symposium on the management of cactus invasions was held in 2015 in Waikoloa Village, Hawaii, as part of the 13th International Conference on Ecology and Management of Alien Plant Invasions (EMAPi [25,26]). As a result of discussions during this symposium, and aiming to collect available information on cactus management worldwide, members of the IOBC GCWG developed a web-based questionnaire in English, French, Italian, Portuguese, and Spanish, and distributed it to all parts of the world known to have invasive cacti (Supplementary Material). Additionally, information was collated from scientific and grey literature, online databases, and scientific reports. The collected information was then synthesised to identify a set of currently available actions to manage the invasions of alien cacti globally. We only considered invasive alien cacti here (i.e. cactus species expanding within their native ranges in the Americas are not discussed).

## 2. Results and Discussion

A total of 95 people from 13 countries / regions (Australia, Austria, France, Italy, Kenya, Lesser Antilles, Macedonia, Mexico, Pacific Islands, Portugal, South Africa, Spain and Tunisia) answered the questionnaire. However, we did not receive any responses from some countries with known cactus invasions, such as Namibia and China. Respondents included alien species managers (38.5%), invasion biologists (27.5%), property owners (8.8%), experts on biological control (7.7%), both professional (6.6%) and amateur (4.4%) horticulturalists, policy makers (4.3%) and food scientists (2.2%).

**Figure 1:**
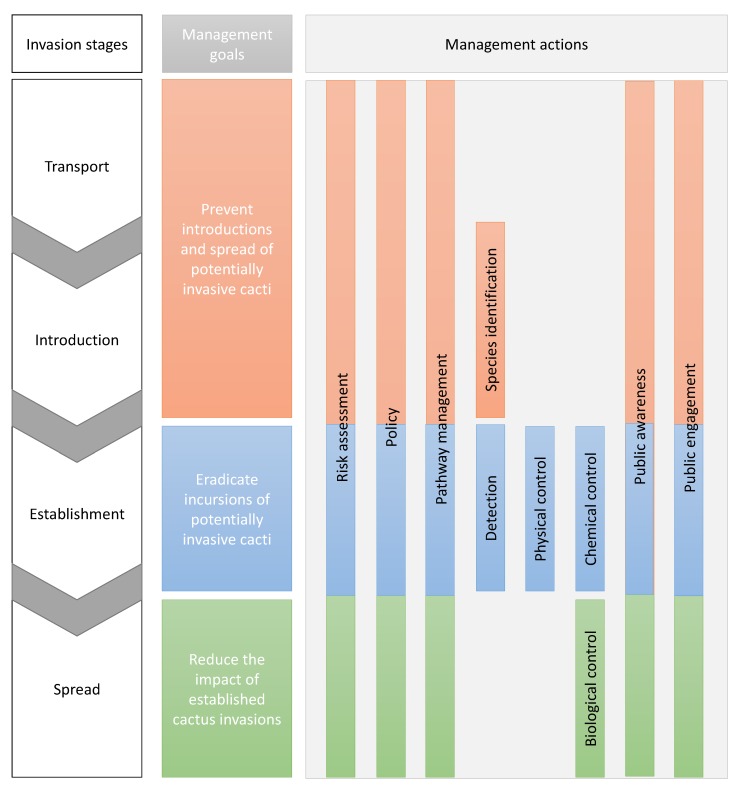
Overview of different actions through which goals of managing cactus invasions can be achieved. Invasion stages are based on the unified framework for biological invasions [37].

Using the information from the questionnaires and the additional sources, we identified 10 management actions, each of which can help achieve one or more of the three main goals of invasive species management (i.e., prevention, eradication, and impact reduction; Figure 1). These are discussed in turn in the sections that follow.

### 2.1. Risk Assessment

Risk assessments for alien species evaluate the likelihood and consequences of alien species becoming invasive. For cacti, most regions use risk assessment schemes targeting alien species in general. These general schemes were the only risk assessment methods reported by the respondents of the questionnaire. The most commonly reported scheme used was the Australian Weed Risk Assessment (A-WRA [27]), which was initially developed for Australia and New Zealand and is currently the most frequently used risk assessment scheme for alien plants [28]. The other main scheme used was the risk assessment protocol developed for central Europe by Weber and Gut [29], and tested in other European regions, such as Spain [30] and France [31].

These general risk assessment schemes assume that a similar set of factors determine the invasion success of all alien species. However, not all species share the same determinants of invasiveness [12]. This has stimulated research on correlates between invasiveness and introduction pathways, species, and habitat characteristics that can potentially predict invasions within particular groups of species, including cacti [15,18,20]. These studies revealed that cacti with detachable segments, spines, and large native range sizes are more likely to become invasive and cause negative impacts, and that most alien cacti can only establish in areas with dry and warm summers [32]. Based on this information, we propose five categories of invasion risk (Table 1): (1) species known to be invasive, (2) species likely to be invasive, (3) species whose invasion is limited by climate, (4) species unlikely to become invasive, and (5) species with no record of invasive behaviour. Cacti classified as species with no record of invasive behaviour are those with long residence times in areas outside their native ranges but no history of invasiveness. For example, the golden barrel cactus (*Echinocactus grusonii* Hildm.), one of the most propagated cactus species worldwide, has never been recorded as invasive [33,34]. Therefore, it meets the criteria of a low risk species that could be included on a permitted or “green” list [35]. In other cases, the evidence for a risk is equivocal. In such instances, it will be important to monitor the introduced populations (if the species is unlikely to become invasive) or conduct more detailed research to quantify the actual risk (if it is likely that its invasion process will be constrained by climate, which is a clear barrier for the establishment of cactus species [32]). For example, more detailed research will be required if *Harrisia martinii* (Labour.) Britton and Rose, a cactus species that is invasive in parts of Australia and South Africa [15,36], was to be introduced to, for example, northern Europe, where the cold, wet climate is likely to prevent invasions. For species that are highly likely to become invasive in the introduced region, introduction should be banned, or if already present, they should be targeted for eradication or containment.

### 2.2. Policy and Legislation

Once species that pose a risk following introduction are identified, it is important to regulate their importation, use and management [33]. According to our survey, the introduction and use of cactus species is regulated in a number of countries (Table 2): 87% of respondents reported regulations concerning the introduction of new alien cacti from other regions and 38% reported regulations concerning the movement of cacti within their region. We crosschecked this information with the Food and Agriculture Organization Legislative and Policy Database (FAOLEX; http://www.fao.org/faolex/). Some of the policies regulate the introduction, use and management of cacti at a national level (e.g., the *South African National Environmental Management: Biodiversity Act 2004* (Act No. 10 of 2004) Alien and Invasive Species Regulations). However, others are more specific, or only act at the state level (e.g., the Biosecurity Act 2015 in New South Wales, Australia). Importantly, cactus nomenclature in the legislation text can be generic, imprecise or out of date. In Table 2, we report the names exactly as they are indicated in the legislation texts.

For regulations to be effective in reducing the threat of biological invasions, they need to be fully implemented, reflecting the interests and enjoying the support of most stakeholders [39]. For example, in South Africa, all major stakeholders directly involved in cactus use, management and policy implementation had a workshop to identify the most feasible approach for regulating cacti introductions and dissemination. Their recommendation was, to a large degree, adopted in the final version of the National Environmental Management: Biodiversity Act, Alien and Invasive Species regulations that came into force in October 2014. This process facilitated the implementation and enforcement of the regulations [33].

### 2.3. Pathway Management

Managing the pathways of alien species introductions and spread is one of the most effective measures that can prevent new invasions occurring. This is particularly effective when a suite of species is predominantly introduced via the same pathways [40]. For pathway management to be effective, it is important to have as much information as possible on the full suite of vectors by which propagules may be introduced [41].

Humans have transported cacti from the Americas to other continents since the 15th century [42]. The earliest reasons for introducing cactus species outside their native ranges were utilitarian—*Opuntia* species were introduced for human consumption, as fodder, for living hedges, and for the production of cochineal [43,44]. While the international trade in cactus species for agricultural uses has declined over time (and only 4% of the people answering the questionnaire mentioned this pathway), the Food and Agriculture Organization of the United Nations (FAO) and the European Union still funds projects to promote the uses of cacti, aiming to reduce the impacts of climate change and land degradation [45]. Mostly spineless varieties of *Opuntia* species, which are believed to be non-invasive [46], are being exported around the world for this purpose [47]. However, both field observations (e.g. in Portugal) and a recent study in South Africa [48] showed that these varieties can revert to spiny forms, so the invasion risk of these varieties needs further research.

The horticultural trade is currently the main reason for the introductions of new cacti: 81% of all cactus species are traded internationally as adult plants or seeds for ornamental purposes [34], and there are hundreds of cactus and succulent ornamental magazines, societies, social media pages and interest groups around the world [15]. This introduction pathway was mentioned by 96% of the respondents of the questionnaire. Once introduced, some cactus species can escape cultivation through a wide variety of pathways, including through the disposal of garden waste and dispersal by domestic animals (including cattle), birds, mammals or reptiles, water, wind, or intentional and unintentional dispersal by humans (e.g., when attached to clothes or vehicles). There are also a number of documented cases where cacti have been deliberately planted in the wild by cacti aficionados with the explicit aim of encouraging them to naturalise, e.g., Essl and Kobler [49].

Introductions of ornamental cacti will therefore need to be prioritised and managed carefully if future invasions are to be avoided. Since only a few of the traded cactus species are invasive or potentially invasive, such management efforts would not result in a substantial restriction of commercial activities [34]. An important component of managing the ornamental pathway of cactus introductions is the voluntary self-regulation of the horticultural trade [50] through the development of codes of conduct. Codes of conduct can encourage nursery owners to stop trading invasive cactus species and to identify native alternatives to invasive ornamental cacti. For example, in 2016, at the Melbourne Gateway Facility, Australia, biosecurity officers using X-ray machines and detector dogs intercepted several packages of potato chips hiding cactus species from Korea. Since then, the Australian Government of the Australian Government has worked closely with eBay and other online stores to educate overseas sellers about Australia’s biosecurity regulations. However, even if voluntary self-regulation of the legal cactus horticultural trade is achieved [34], the large illegal horticultural trade [51,52] still needs to be regulated and monitored, since some specialist collectors of cacti are prepared to break the law to access new species for their collections. Managing e-commerce will be an on-going challenge.

### 2.4. Species Identification

Limiting the introduction of potentially invasive cactus species necessitates the availability of accurate tools to identify taxa during regular inspections at ports of entry [53]. The identification of cacti is, however, very challenging [32]. There is substantial nomenclature instability within the family, possibly for some fundamental evolutionary reasons, but also for a couple of practical reasons: cacti are poorly represented in herbaria as they are difficult to collect and curate [19], and flowers and fruits are often needed for identification, but some species take years to flower [32]. As a result, they are often listed in the literature and the ornamental trade under incorrect names [34]. DNA barcoding can be used to identify seeds and adult plant introductions of cacti [54], but it is costly and currently impractical given the scale of the horticultural trade, and, due to gene conservatism in the Cactaceae, not all species can be identified to species or even genus level [34,55,56].

An alternative tool for identifying high risk introductions of cactus seeds is the use of seed size and seed mass as indicators. Novoa et al. [57] showed that invasive species have significantly bigger and heavier seeds than non-invasive species. Therefore, although these traits are probably not relevant for the invasiveness of cactus species (i.e., invasive cacti disperse mainly through their detachable fragments [15,32]), they could be used as indicators to detect unwanted introductions. Moreover, when introduced as adult plants, high risk cacti introductions can also be identified by their growth form (i.e., cacti with flattened-padded and angled growth forms are more likely to become invasive and cause an impact [15,20]).

### 2.5. Detection of New Incursions

For the successful eradication of any invasive species, new incursions must be promptly detected and delimited. Actions to detect alien cacti are in place in several regions of the world, and 80% of the respondents of the questionnaire mentioned some. For example, the Southern African Plant Invaders Atlas has recorded the presence of alien plants (including cacti) growing outside of cultivation since 1994 and has recently focused efforts on finding populations of potentially eradicable species [58]. In 2008, the government of Valencia (Spain) established an alert network of 242 forest wardens and other trained staff to detect *Cylindropuntia rosea* (DC.) Backeb. [59]. Within two years, the network detected 38 new invasions. In Kruger National Park (≈1.9 million ha), rangers record alien plant observations in the course of their daily patrols using customised CyberTracker software [60,61]. This has enabled the detection of new populations of *O. stricta* in different areas of the park, and changes in the distribution and abundance of *O. stricta* across the 67,000 ha management units. Cactus invasions are often suitable targets for detection using remote sensing techniques because they primarily invade arid regions or dry habitats and are often the only green components of vegetation, especially during the dry season [62]. However, detecting low-density populations of invasive cacti is still a major challenge (e.g., a test of high-resolution light detection and ranging remote sensing (LIDAR) revealed its inability to detect low-density invasive populations of *O. stricta* in Kruger National Park).

In recent years, citizen science has become an increasingly popular tool to assist with the detection and mapping of alien species [63,64,65]: citizen science projects were mentioned by 27% of the respondents to the questionnaire. For example, the Department of Agriculture and Food of Western Australia (DAFWA) developed MyWeedWatcher, an application that allows volunteer users to report the presence of non-native species, including cacti (https://www.agric.wa.gov.au/myweedwatcher). Citizens also use the Facebook group “Weeds of Western Australia” to post new detections of alien cacti. In Queensland, Australia, the Weed Spotters Network Queensland aims to detect new invasions of potential weeds, including cacti (https://www.qld.gov.au/environment/plants-animals/plants/herbarium/weeds/weed-spotters). In Portugal, volunteer users record the sightings of alien species, including cacti, in the online and Android platform invasoras.pt (http://invasoras.pt [66]), and in southern Africa, new records of cactus invasions have also been detected by volunteer users through uploading geo-referenced photographs to the online site iNaturalist (https://www.inaturalist.org/places/south-africa). Experts can then scrutinise the photographs on-line to validate or determine the species identity. This is particularly necessary when detecting populations of potentially invasive cacti since their identification is difficult [32]. Even so, identification to the species level based only on photographs is not easy, and as such, for example, at invasoras.pt *Opuntia* species are validated only to the genus level [66].

### 2.6. Physical Control

In our questionnaire, several physical control methods were reported as being used to manage cactus invasions. Such methods involve the physical removal of plants, using bulldozers, digging hoes, excavators, shovels, spades or rakes, followed by treatment and burial. Options listed by the respondents for treating the removed plants before burying them include placing them in water for a minimum of 16–20 days (to promote rotting), or drying or burning them at a minimum of 2 m above the surface, to avoid reshooting. Due to the ability of most cactus species to reproduce both sexually (i.e., from seeds that are generally dispersed by birds or mammals) and vegetatively (i.e., from any small fragment that might remain after physical clearing), treated areas need to be monitored for several years to detect regrowth and achieve complete eradication. Moreover, all equipment, including machinery, should be checked for any attached seeds or fragments to avoid the dispersion of the invaders elsewhere [67].

Since physical removal requires substantial funding capacity (e.g., US$540/hectare in Kenya) and time, it is only used for cactus invasions that cover small areas [68]. However, physical control methods were the methods most frequently reported by the respondents (i.e., 79% of the respondents mentioned physical methods, while 62% and 45% mentioned chemical and biological methods, respectively).

In addition to physical removal, cactus invasions can be contained by preventing the movement of cactus cladodes and seeds dispersers. For example, a landowner in Longreach, western Queensland, aiming to contain the vegetative spread of *Cylindropuntia fulgida* var. *mamillata*, erected a fence to stop the movement of emus, kangaroos and livestock through its property, since they disperse the cladodes of *C. fulgida* var. *mamillata*.

### 2.7. Chemical Control

The use of chemical products, such as herbicides, is usually more cost-efficient than physical methods for managing cactus invasions [59,68]. A wide range of herbicides can be used to manage invasive cacti (Table 3). For example, in Australia, the herbicides with the active ingredients Amitrole, Monosodium methyl arsenate (MSMA), Triclopyr, and Triclopyr + Picloram are registered for the management of cactus invasions (www.apvma.gov.au). However, other countries have different regulations regarding the use of herbicides. For example, a number of herbicides used in Australia and South Africa to control *Cylindropuntia rosea* are not allowed to be used in Europe [59], or cannot be used in protected areas or in the proximity of water bodies.

Herbicides can be applied to cacti as a foliar spray or through stem injections. The advantages of stem injections are that systemic chemicals are rapidly translocated to all parts of the plant, they cause minimal damage to non-target species and costs are lower. However, in certain thicket-forming and spiny cacti, access to the stems can be difficult [69] and dangerous due to the spines. In such cases, herbicides can be applied through a foliar spray. When applying a foliar spray, it is important to cover all parts of the plant with the herbicide as translocation of the active components among segments is very low. To achieve this goal, it is extremely useful to add a dye to the mixture being sprayed so that no plant parts are missed or sprayed twice, making herbicide application less time consuming and more efficient. Moreover, the epidermis of cacti is covered by a thick protective waxy layer, which together with the Crassulacean Acid Metabolism (CAM) photosynthetic pathway (i.e., the stomata are closed during daytime) can severely restrict the uptake of herbicides [68]. To overcome this, herbicide application should be combined with the use of effective wetting agents [70]. Herbicides (including both surface spray and stem injection techniques) are preferably applied when air temperatures do not exceed 30 °C as extreme heat, cold or drought conditions encourage plant dormancy, which may reduce chemical uptake.

For widespread cactus invasions, physical and chemical control are expensive and require extensive follow up efforts [68]. Several chemical campaigns have failed to control large cactus invasions due to the high costs involved and the rapid recovery of the populations e.g., [71,72,73]. Moreover, the application of herbicides can pose a risk to humans and the environment. For example, the herbicide glyphosate has been used for weed control since the 1970s. However, in March 2015, the World Health Organization’s International Agency for Research on Cancer classified glyphosate as “probably carcinogenic to humans” [74]. Therefore, chemical techniques are generally only recommended to manage small (e.g., < 1 ha) to medium (1–10 ha) invasions, or to manage key sites within the larger invaded areas.

### 2.8. Biological Control

Biological control (biocontrol) is the most cost-effective option for managing widespread cactus invasions, and it has been extremely successful in some regions [75,76,77]. The native range of cacti is restricted to the Americas [32], and most natural enemies of Cactaceae have host ranges restricted to the family and are therefore, appropriate for use as biocontrol agents outside of the New World [78,79]. Biological control of cacti has been practiced for over 100 years in several countries, using multiple natural enemy species (Table 4). Despite the large number of biocontrol agents introduced and the length of time the agents have been present in their introduced range, there have been no recorded non-target impacts outside the New World [68]. However, the use of biological control in the Americas (i.e., to manage a cactus species introduced from another part of the Americas), must use only agents that are much more host-specific so as not to harm any native cactus species.

The first successful biological control project targeting cactus species dates back to 1913, when the cochineal *Dactylopius ceylonicus* (Green) (Hemiptera: Dactylopiidae) was introduced to South Africa to control the invasive *Opuntia monacantha* [80,81]. Due to its effectiveness, *D. ceylonicus* was subsequently introduced successfully to La Réunion, Mauritius and Australia. In the 1920s, the agents *Dactylopius opuntiae* (Cockerell) and *Cactoblastis cactorum* (Berg.) (Lepidoptera: Pyralidae) were released in Australia to control the invasion of *O. stricta* [82]. This project was extremely successful, largely due to extensive release efforts made by the Commonwealth Prickly Pear Board, state government officials and affected landholders [83]. For example, Raghu and Walton [22] calculated that by 2005, the investment of $21.1 million returned a value of $3110.3 million. The return on investment for other cactus biocontrol programmes have also been very favourable, such as the programme against *Opuntia aurantiaca* Lindl. in South Africa, which is estimated to have saved the country ZAR 6.1 billion with a benefit/cost ratio of 709:1 [84]. The success of these projects increased interest in the biological control of invasive cacti.

**Figure 2:**
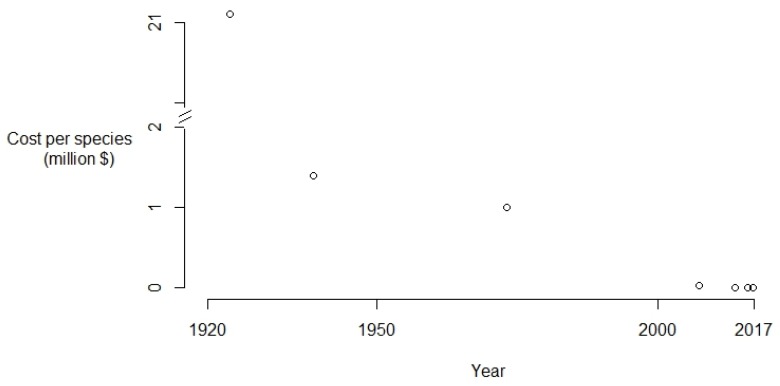
Costs involved in the development and initial implementation of biological control actions for invasive cactus species. US$ are adjusted for 2018 values. Each point corresponds to a different biological control campaign, in chronological order: *Opuntia stricta* in Australia [82], *O. aurantiaca* [84], *Harrisia martinii* [90], *Cylindropuntia fulgida* var. *fulgida* [91], *O. humifusa* [90], *C. fulgida* var. *mamillata* [91] and *H. pomanensis* [90] in South Africa.

Since these early successes, biological control has been used to manage another 34 invasive cactus species (Table 3). For example, the control of *Opuntia ficus-indica* (L.) Miller in South Africa, which has been permanently reduced from a distribution covering 900 000 ha to only 100 000 ha, and the control of *O. stricta* in the Kruger National Park of South Africa where densities of the plant were reduced by over 90% [23,87] (Box 1). These transfers of successful biocontrol agents from one country to another (so called “piggy-back” projects) are text-book cases of the benefits of sharing management experiences [88]. Moreover, some of the biocontrol agents introduced to control cacti are effective against more than one invasive cactus species (Table 3), resulting in a substantial reduction in the cost of developing and testing biocontrol agents for other cactus invaders. For example, *Opuntia humifusa* (Raf.) Raf. was shown to be susceptible to the “stricta” biotype of *D. opuntiae* introduced in South Africa to control the invasion of *O. stricta* [89]. Also, the biological control agent *Hypogeococcus festerianus* (Lizer y Trelles) (Hemiptera: Pseudococcidae), which was initially introduced into Australia and South Africa to control *H. martinii*, now also plays a role in the control of *H. pomanensis*, *H. tortuosa* and *Cereus jamacaru* DC [75,90]. It was also recently released against *Cereus hildmannianus* K.Schum subsp. *uruguayensis* in Australia to help with managing this species. This, together with the lessons, approaches and techniques learned, have reduced the costs of developing new biological control agents for cacti. Therefore, these techniques are becoming more cost-effective, with increasing benefit/cost ratios over time (Figure 2). The use of biological control for widespread and abundant invasive alien cactus species should therefore be promoted. When effective biological control agents for a species exists elsewhere in the world, they should be shared with countries that have the same cactus invasions [89]. New biocontrol agents should only be developed for cactus species that are widespread and problematic but do not have effective agents available.

### 2.9. Public Awareness

Public awareness campaigns can aid management by informing the public about the invasion risk and impacts of invasive cacti [34]. The results of our questionnaire survey show that several tools have already been used to raise awareness regarding cactus invasions, including books, e.g., Walters et al. [19]; documentaries (e.g., https://www.youtube.com/watch?v=K9THmDhhA4A); Facebook groups (e.g., https://www.facebook.com/groups/918877938264005/); fact sheets (e.g., https://www.cabi.org/Uploads/CABI/news/Cactus-Factsheet.pdf); newsletters (e.g., http://invasives.org.za/resources/sapia-news); public talks to NGOs, private or public environmental managers, school learners or universities; video interviews (e.g., https://www.youtube.com/watch?v=kLab381i95U); voluntary activities; and websites (e.g., http://www.aicn.org.au/). For example, Marchante and colleagues developed a website (http://www.invasoras.pt) in 2003, updated in 2013, with information on the invasive plants of Portugal, including several cactus invaders. By 2019, the website had been accessed by more than 390,200 visitors (E. Marchante, personal communication), is connected to a citizen-science platform for the mapping of invasive plants and to several social media and other awareness initiatives and was validated as an effective awareness tool [92]. The citizen-science platform received more than 750 reports of sightings of *Opuntia maxima* Miller (or very similar species), by almost 90 users, showing some awareness of this species as being invasive in the country. Another example is the manual developed in 2017 by the Western Australian Department of Primary Industries and Regional Development (DPIRD) and Biosecurity of South Australia, aiming to help industry and land managers to manage cactus invasions [93].

Box 1 ***Opuntia ficus-indica* control by *Dactylopius opuntiae* in Spain.**

*Dactylopius opuntiae* is an insect able to control the invasion of *O. ficus-indica*. *Dactylopius opuntiae* was introduced to Spain by unknown routes. It was first detected in Hellín (Murcia, East Spain) in 2007. However, it soon experienced vertiginous expansion, owing to the almost continuous distribution of the host plant *O. ficus-indica* along the Mediterranean coast, and passive undirected dispersal (mainly by wind) of the cochineal. Recent studies in Valencia (East Spain) revealed that *D. opuntiae* can disperse up to 2 km in 16 months. Currently, the cochineal is found from Huelva to Barcelona and has caused a massive decline of Spanish *O. ficus-indica* invasive populations. The injury of *D. opuntiae* to plants takes months to manifest, initially as turgor loss of cladodes followed by chlorosis and subsequent collapse. The death of plants might take years and is dependent on local climatic conditions, with plants colonising xeric environments being more vulnerable to cochineal attack than those growing on more mesic sites. We hypothesise that the colonisation of *D. opuntiae* will lead to overall control of *O. ficus-indica* expansion in Spain and to localised extinctions, depending on climatic conditions. However, *D. opuntiae* is also impacting commercial plantations of spineless *O. ficus-indica,* which is driving the demand of commercial growers in Spain and Portugal for the introduction of natural enemies of the cochineal. However, if natural enemies are released as biocontrol agents for cochineal, they will not be restricted to commercial plantations and it is very likely that the level of control of invasive populations of *O. ficus-indica* will be substantially reduced. It is also possible that such agents could spread naturally to other regions, threatening the successful biological control of cactus invasions across Africa. For this reason, we think it is very important to engage with farmers cultivating *O. ficus-indica* regarding the costs of invasions and the use of best practices to avoid the spread of the plant from cultivated land and to promptly remove any wildings. Moreover, research should promote the development of less invasive genotypes, e.g., seedless fruits or sterile cultivars.

### 2.10. Public Engagement

Public engagement can help to integrate the knowledge and perspectives of different stakeholders in the design and implementation of effective management actions and to deal with potential conflicts of interest [94]. For example, in South Africa, some invasive cacti (e.g., *O. ficus-indica*) have had a long history of both socio-economic benefits and negative environmental and socio-economic impacts. Through an engagement process including those stakeholders who benefit from cacti in South Africa and those who bear the costs of the invasion, Novoa et al. [18] enabled the participation of all stakeholders in the design of actions to manage cactus invasions in South Africa and helped minimise conflicts by clarifying stakeholder’s beliefs and exploring consensus solutions. In Europe, cacti invasions have been promoted by the (often illegal) planting of cacti in the wild by succulent aficionados [49]. Moreover, in Portugal, there is a conflict of interest between conservationists (who consider *O. ficus-indica* as invasive) and growers (who are discussing the need to explore possible biocontrol agents to control the biocontrol agent accidentally introduced in Spain to manage *O. ficus-indica* invasions (Box 2)). There is thus an urgent need to engage with cacti growers and horticultural societies and to raise awareness on the risks caused by alien cacti.

Box 2 ***Opuntia stricta* invasion in Kruger National Park, South Africa.**

**Figure 3:**
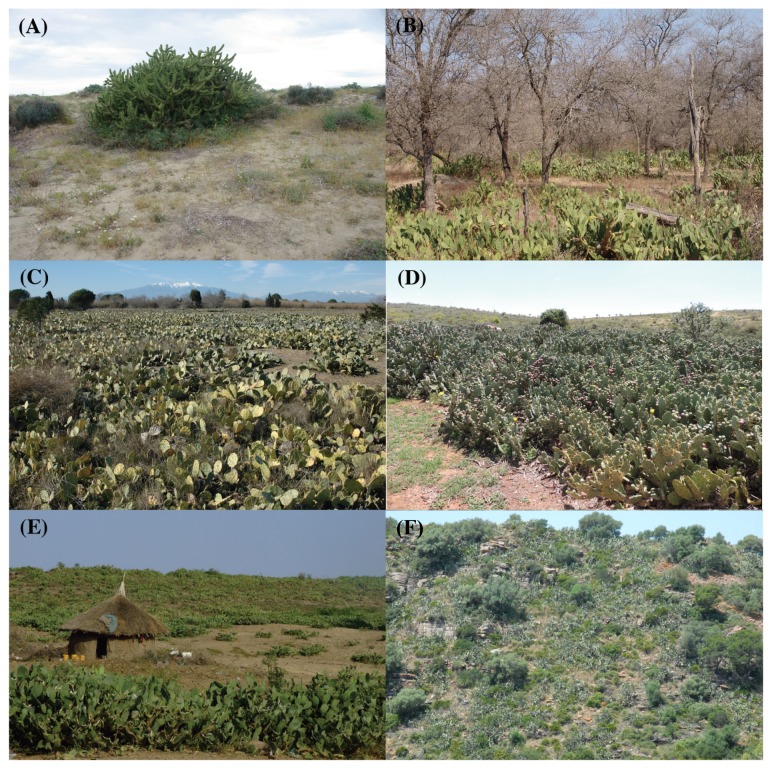
Examples of cactus invasions in different parts of the world. (**A**) *Austrocylindropuntia* sp. invading sand dunes in Sardinia, Italy. *Opuntia stricta* invading (**B**) a semi-arid savanna in Kruger National Park, South Africa, (**C**) grey dunes in southern France and (**D**) a rural area in Kenya. *Opuntia ficus-indica* invading (**E**) a village in Ethiopia and (**F**) Calderona Natural Park in Valencia, Spain.

*Opuntia stricta* was introduced into Skukuza staff village in Kruger National Park (KNP), South Africa, in the mid-1950s, reportedly as an ornamental plant. Dispersal by elephants, baboons and birds, meant that by 1980, about 1000 ha were reported to be invaded (Figure 3). In 1987, chemical control efforts commenced, and the biological control agent *Cactoblastis cactorum* was released in 1989. However, these management efforts were not highly successful, and by 1996, 30,000 ha were reported to be invaded. In 1997, the “stricta” biotype of *D. opuntiae* was released and within six years, the biomass of *O. stricta* declined by about 90%, remaining at low densities ever since. Due to the combined effect of *D. opuntiae* and *C. cactorum*, nearly all fruiting plants were destroyed, limiting further long-distance dispersal. Due to this success, biocontrol was planned within the containment area (≈67,000 ha) and a mass-rearing facility constructed to disperse the agents within the KNP and its surrounding areas. New populations detected by rangers in their daily patrols elsewhere in the park (e.g., Olifants and Letaba Rivers) are extirpated physically or chemically soon after detection.

## 3. Conclusions

In conclusion, we (as the IOBC GCWG) recommend that:

(1) The trade in invasive and potentially invasive cactus species SHOULD BE regulated, taking into consideration their classification in five broad risk categories: known threat, likely threat, invasion limited by climate, invasion unlikely, and species with a track record of no invasions (Table 1). This is possible as the ornamental trade is currently by far the major pathway for the introduction of new cactus species to areas outside their native range. Moreover, invasiveness in the Cactaceae appears to vary predictably, such that certain traits are of sufficient diagnostic value to allow for the identification and flagging of high-risk species (e.g., at ports of entry).
(2) National strategies and action plans consider developing efforts to detect cactus invasions early in the invasion process and eradicate them using physical or chemical control before they become widespread. The use of herbicide is often the most cost-effective management option for such small and localised populations.
(3) Managers with land with extensive and/or abundant cactus invasions should use biological control agents to reduce the abundance, density and impacts of widespread cactus invasions in a manner sensitive to native species and the needs of cactus users.
(4) Policies and actions are implemented to promote public awareness and engagement activities to integrate available knowledge and all perspectives in the process of developing and implementing management actions, and to deal with potential conflicts of interest, especially in the agricultural and ornamental horticulture sectors.

Achieving these four recommendations will require global co-operation facilitated, encouraged and supported by the IOBC GCWG, and the dissemination of information and experience.

Developing management actions for groups of species with similar management requirements, in collaboration with stakeholders from different invaded areas, is an effective way of avoiding duplicating research efforts and ensuring the cost-effective allocation of management resources [95,96]. This is the case for invasive cacti, for which a large body of research and information is available in different regions of the world. This can provide a reasonable understanding of what management actions are available, and where there is a well-documented history of management implementation. Unfortunately, this is not the case for many other groups of alien species requiring management.

We believe that applying the approach of grouping species with similar management requirements and linking practitioners, researchers and managers working on such species in different regions will help to identify more accurate, efficient and transferable options for managing invasive species in the future. Therefore, we suggest that creating “global networks for invasion science” as proposed by Packer et al. [97] is a valuable approach and the results presented here, through the efforts of the Global Cactus Working Group, illustrates how effective this approach can be.

## Figures and Tables

**Table 1:** Proposed scheme for categorising cactus species based on the risks that they will become invasive and cause negative impacts in a given introduced range (based on an approach developed for Australian acacias [38]).

Categories	Criteria	Recommendations
Known threat	Species is known to be invasive AND The introduced range has a suitable climate	Ban introductions Target taxa for surveillance Target individuals and existing populations for eradication or control
Likely threat	Species is not currently recorded as invasive AND Species has detachable segments and/or spines and a large native range AND The introduced range has a suitable climate	Ban introductions Target taxa for surveillance Target individuals and existing populations for eradication or control
Invasion limited by climate	[Species is known to be invasive AND/OR Species has detachable segments and/or spines and a large native range] AND The introduced range does not have a suitable climate	Conduct detailed research Monitor existing individuals and/or populations for spread Include planting sites in a network of sentinel gardens
Invasion unlikely	Species is not currently recorded as invasive AND Species does not have detachable segments and/or spines or does not have a large native range AND The introduced range does not have a suitable climate	Allow introduction Monitor existing individuals and/or populations for spread Include planting sites in a network of sentinel gardens
Species with a track record of no invasions	Species is not currently recorded as invasive despite having been planted in climatically suitable introduced ranges for over 50 years	Allow introduction elsewhere/add to a permitted list

**Table 2:** Examples of policies regulating the introduction and use of cactus species in a number of countries.

Country	Area	Taxa Regulated (Name as in the Text)	Policy	Notes
Australia	National	*Opuntia* spp. (with the exception of *O. ficus-indica*), *Cylindropuntia* spp. and *Austrocylindropuntia* spp.	Weeds of National Significance (WoNS)	Indicates that the state and territory governments are responsible for their legislation, regulation and administration
Australia	Queensland	*Opuntia* spp. (with the exception of *O. ficus-indica*), *Cylindropuntia* spp. and *Austrocylindropuntia* spp.	Land Protection (Pest and Stock Route Management) Act 2002	Regulates their introduction, use and management
Australia	New South Wales	*Opuntia* spp. (with the exception of *O. ficus-indica*), *Cylindropuntia* spp. and *Austrocylindropuntia* spp.	Biosecurity Act 2015	Regulates their introduction, use and management
Australia	Northern Territory	*Opuntia* spp. (with the exception of *O. ficus-indica*), *Cylindropuntia* spp. and *Austrocylindropuntia* spp.	Weeds Management Act 2013	Regulates their introduction, use and management
Australia	South Australia	*Opuntia* spp. (with the exception of *O. ficus-indica*), *Cylindropuntia* spp. and *Austrocylindropuntia* spp.	Natural Resources Management Act 2004	Regulates their introduction, use and management
Australia	Victoria	*Opuntia* spp. (with the exception of *O. ficus-indica*), *Cylindropuntia* spp. and *Austrocylindropuntia* spp.	Catchment and Land Protections Act 1994	Regulates their introduction, use and management
Australia	Western Australia	*Opuntia* spp. (with the exception of *O. ficus-indica*), *Cylindropuntia* spp. and *Austrocylindropuntia* spp.	Biosecurity and Agricultural Management Act 2007	Regulates their introduction, use and management
Botswana	National	*Opuntia aurantiaca* and *Opuntia imbricata* (Haw)	Noxious Weeds Order (Chapter 35:04). Consolidated version of S.I. No. 49 of 1968 as at 31 December 2013 and amended by S.I. No. 84 of 1976	Declared as noxious weeds
Kenya	National	*Opuntia inermis* and *Opuntia stricta*	Plant Protection Order, 1961. Consolidated version of 2012 of L.N.744/19661 as amended last by L.N. 130/1990	Declared as pest plants
Italy	Tuscany region	*Opuntia ficus-indica*	Regional Act No. 56 making provision for the conservation and the protection of natural and seminatural habitats, flora and wildlife and laying down amendments to Regional Act No. 7 of 23 January 1998 and to Regional Act No. 49 of 11 April 1995.	*Opuntia ficus-indica* cannot be planted in the natural environment
Portugal	National	All cacti except *Opuntia ficus-indica* (L.) Miller	Decree-Law No. 565/99 regulating the introduction of exotic flora and fauna species	States that the regulated species cannot be introduced without undertaking a detailed risk assessment showing the lack of risk of invasion
Portugal	National	*Opuntia elata* Salm - Dyck, *Opuntia maxima* Miller and *Opuntia subulata* (Muehlenpf.) Engelm (= *Austrocylindropuntia subulata*) are listed as invasive (National List of Invasive Species) in the Mainland and Madeira and Azores Archipelagos; *Opuntia tuna* (L.) Mill. is listed only in Madeira. *Opuntia ficus-indica* (L.) Miller is listed under an exception regime whereby growers and nursery workers must comply with the duties of care and reporting, as well as control plans. The introduction into the wild of any other cactus species is subject to authorisation by the nature conservation authority (ICNF).	Decree-Law No. 92/2019 regulates the control, keeping, introduction into nature and restocking of exotic species and implementation at the national level of Regulation (EU) No. 1143/2014, on the prevention and management of the introduction and spread of invasive alien species.	Species listed as invasive, except *Opuntia ficus-indica*, which is included in an exceptional regime, cannot be detained, cultivated, traded, introduced into the wild and repopulated.
South Africa	National	Thirty-five cactus species are regulated as invasive in the country [33], as well as most of their congeners	National Environmental Management: Biodiversity Act: List of invasive species (No. 599 of 2014). It implements the National Environmental Management Biodiversity Act, 2004 (No. 10 of 2004). 2004-05-31 “Alien and Invasive Species Regulations”	Regulates their introduction and use
Spain	National	*Cylindropuntia* spp., *Opuntia dillenii* (Ker-Gawler) Haw *Opuntia maxima* Miller., and *Opuntia stricta* (Haw.)	Real Decreto No. 630/2013 - Regula el Catálogo español de especies exóticas invasoras.	Regulates their introduction and use
Uganda	National	*Opuntia* spp.	Plant Protection (Importation of Plants) Order (S.I. 31-3); consolidated version of Statutory Instrument 3 of Cap. 31, History: S.I. 244-3	Prohibited plants and seeds
USA	National	*Opuntia aurantiaca* Lindley	Noxious Weed Regulations (7 CFR 360.100-360.600); consolidated version as at 1 January 2018	Designated as a noxious weed (to prevent their introduction into the United States or their dissemination within the United States).
Vanuatu	National	*Opuntia* spp.	Prevention of Spread of Noxious Weeds Act (Cap. 44).	Declared as a noxious weed
Zambia	National	*Opuntia* spp., including spineless cactus, vegetative material, seed and fruit of, for propagation	Plant Pests and Diseases (Importation) Regulations (Cap. 233); consolidated version of F.G.N. No. 144 of 1960 as at 2006 and amended last by G.N. No. 497 of 1964	These Regulations provide for the control of the importation of plants and related items for purposes of plant health. It puts a total ban on *Opuntia* spp.
Zimbabwe	National	*Opuntia aurantiaca* Lindl.	Environmental Management Act (Chapter 20:27); consolidated version of Act No. 13 of 2002 as amended by Act No. 6 of 2005 with effect as from 1 July 2005	Declared as a noxious weed

**Table 3:** Herbicides listed by questionnaire respondents and management reports as effective for managing cactus invasions. It is important to note that the use of some of the listed herbicides might be banned or restricted in certain countries or in protected areas.

Herbicide	Concentration of the Active Ingredient	Dilution	Application	Notes
Amitrole	250 g/L amitrole and 220 g/L ammonium thiocyanate	1:25 in water	Foliar spray	Expensive but efficient
Glyphosate	450 g/L glyphosate	1:3 in water	Stem injection	Inject 4 mL per cladode
Metsulfuron-methyl	600 g/L metsulfuron-methyl	0.03:100 in water	Foliar spray	
MSMA	800 g/L MSMA	2.5:100 in water	Foliar spray	
		No dilution	Stem injection	Inject 4 mL per meter of plant height per stem branch
Triclopyr	600 g/L Triclopyr	3:100 in water or 1.5:100 in diesel	Foliar spray	The diesel mix often yields better results
Triclopyr and Picloram	240 g/L Triclopyr and 120 g/L Picloram	1:60 in diesel	Foliar spray	
Triclopyr and Picloram	300 g/L Triclopyr and 100 g/L Picloram	1:100 in water	Foliar spray	
Triclopyr, Picloram and Aminopyralid	300 g/L Triclopyr, 100 g/L Picloram and 9g/L Aminopyralid	1:100 in water	Foliar spray	
Triclopyr and Fluroxipir	30 g/L Fluroxipir + 90 g/L Triclopir	1:100 in water	Foliar spray	Efficiency declines with cactus size. Requires several applications for a complete kill of large specimens. Expensive.

**Table 4:** A list of all biocontrol agents that have been released against invasive alien cacti based on Klein [85], Winston et al. [21], Zimmermann [68] and Zachariades [76,77], as well as biological control practitioners. The feeding guilds are classified *sensu* Barbetta [86]. Establishment is categorised as “Yes”, “No” or “Under investigation” depending on whether there is evidence of a self-perpetuating population of the agent after release or on whether this evidence is still under investigation. The severity of damage is rated *sensu* Olckers and Hill [80] as extensive (very high levels of damage, as much as could be expected from the agent; few plants survive, or growth is arrested, or almost no seeds are produced), considerable (high levels of damage; some plants may survive but growth rates are noticeably slower, or seed production is reduced by more than 50%), moderate (perceivable damage, but most plants survive; growth may be slowed to some extent, or seed production is reduced by less than 50%), trivial (some damage, but survival, growth and seed production of the plants is almost normal), none (no damage) or unknown (agent has been too recently released, or has not been evaluated yet).

Host Species	Biocontrol Agent	Feeding Guild	Establishment	Country of Release	Severity of Damage to the Host Plant
*Acanthocereus tetragonus* (L.) Hummelinck	*Hypogeococcus festerianus* (Lizer y Trelles)	Stem sucker	Yes	Australia	Moderate
	*Nealcidion cereicola* (Fisher)	Stem borer	No	Australia	NA
*Austrocylindropuntia subulata* (Muehlenpf.) Backeb	*Cactoblastis cactorum* (Berg)	Cladode borer	Yes	South Africa	Unknown
*Cereus hildmannianus* K. Schum.	*Hypogeococcus festerianus* (Lizer y Trelles)	Stem sucker	Yes	South Africa	Extensive
	*Nealcidion cereicola* (Fisher)	Stem borer	Yes	South Africa	Considerable
*Cereus hildmannianus* K. Schum subsp. *Uruguayensis*	*Hypogeococcus festerianus* (Lizer y Trelles)	Stem sucker	Yes	Australia	Too early to determine
*Cereus jamacaru* DC.	*Hypogeococcus festerianus* (Lizer y Trelles)	Stem sucker	Yes	South Africa	Considerable
	*Nealcidion cereicola* (Fisher)	Stem borer	Yes	South Africa	Considerable
*Cylindropuntia fulgida* (Engelm.) F.M. Knuth var. *fulgida*	*Dactylopius tomentosus* (Lamark), “*cholla*” biotype	Cladode sucker	Yes	South Africa Zimbabwe	Extensive
	*Dactylopius tomentosus* (Lamark), “*imbricata*” biotype	Cladode sucker	Yes	South Africa	Trivial
*Cylindropuntia fulgida* (Engelm.) F.M. Knuth var. *mamillata*	*Dactylopius tomentosus* (Lamark), “*cholla*” biotype	Cladode sucker	Yes	Australia Namibia South Africa Zimbabwe	Extensive
*Cylindropuntia imbricata* (Haw.) F.M. Knuth	*Cactoblastic cactorum* (Berg)	Cladode sucker	Yes	South Africa	Trivial
	*Dactylopius tomentosus* (Lamark), “*imbricata*” biotype	Cladode sucker	Yes	Botswana Namibia South Africa	Considerable
				Australia	Considerable
	*Dactylopius tomentosus* “*cylindropuntia sp*.” biotype	Cladode sucker	Unknown	Australia	Too early to determine
	*Metamasius spinolae* (Gyllenhal)	Stem borer	No	South Africa	-
*Cylindropuntia kleiniae* (DC.) F.M. Knuth	*Dactylopius tomentosus* (Lamark), “*imbricata*” biotype	Cladode sucker	Yes	Australia	Considerable
*Cylindropuntia leptocaulis* (DC.) F.M. Knuth	*Dactylopius tomentosus* (Lamark), “*imbricata*” biotype	Cladode sucker	Yes	Australia	Considerable
				South Africa	Extensive
*Cylindropuntia rosea* (DC.) Backeb = *Cylindropuntia pallida* (Rose) F.M. Knuth	*Dactylopius tomentosus* (Lamark), “*imbricata*” biotype	Cladode sucker	Yes	Australia	Trivial
	*Dactylopius tomentosus “califórnica* var. *parkeri” biotype*	Cladode sucker	Yes	Australia	Too early to determine
*Cylindropuntia prolifera*	*Dactylopius tomentosus “califórnica* var. *parkeri”* biotype	Cladode sucker	Yes	Australia	Too early to determine
*Cylindropuntia spinosior*	*Dactylopius tomentosus “bigelovii”* biotype	Cladode sucker	Unknown	Australia	Too early to determine
*Harrisia balansae* (K. Schum.) N.P. Taylor & Zappi = *Harrisia bonplandii* (Pfeiff.) Britton and Rose	*Hypogeococcus festerianus* (Lizer y Trelles)	Stem sucker	Yes	South Africa	Considerable
*Harrisia martinii* (Labour.) Britton and Rose	*Eriocereophaga humeridens* O’Brien	Cactus feeder	No	Australia	-
	*Hypogeococcus festerianus* (Lizer y Trelles)	Stem sucker	Yes	Australia South Africa	Considerable
	*Nealcidion cereicola* (Fisher)	Stem borer	Yes	South Africa	Moderate
*Harrisia pomanensis*	*Hypogeococcus festerianus* (Lizer y Trelles)	Stem sucker	Yes	South Africa	Considerable
*Harrisia regelii* (Weing.) Borg	*Hypogeococcus festerianus* (Lizer y Trelles)	Stem sucker	Yes	Australia	Considerable
	*Nealcidion cereicola* (Fisher)	Stem borer	Yes	Australia	None
*Harrisia tortuosa* (J. Forbes ex Otto and A. Dietr.) Britton and Rose	*Hypogeococcus festerianus* (Lizer y Trelles)	Stem sucker	Yes	Australia South Africa	Considerable
	*Nealcidion cereicola* (Fisher)	Stem borer	Yes	Australia	None
*Hylocereus undata* (Haw.) Britton and Rose	*Hypogeococcus festerianus* (Lizer y Trelles)	Stem galler	Unknown	South Africa	Unknown
*Opuntia aurantiaca* Lindl.	*Cactoblastis cactorum* (Berg)	Cladode sucker	Yes	Australia South Africa	Moderate
	*Dactylopius austrinus* De Lotto	Cladode sucker	Yes	Australia South Africa	Considerable
	*Dactylopius ceylonicus* (Green)	Cladode sucker	No	Australia	-
	*Melitara prodenialis* Walker	Cladode borer	No	Australia	-
	*Mimorista pulchellalis* Dyar	Cladode borer	No	South Africa	-
	*Nanaia* sp.	Cladode borer	No	South Africa	-
	*Zophodia tapiacola* (Dyar)	Cladode borer	Yes	Australia	Moderate
			No	South Africa	-
	*Tucumania tapiacola* Dyar	Cladode borer	Yes	Australia	Trivial
			No	South Africa	-
*Opuntia elata* Link and Otto ex S-D	*Dactylopius ceylonicus* (Green)	Cladode sucker	Yes	Australia	Trivial
*Opuntia elatior* Mill.	*Dactylopius ceylonicus* (Green)	Cladode sucker	No	India	-
	*Dactylopius opuntiae* (Cockerell)	Cladode sucker	Yes	India Indonesia	Extensive
*Opuntia engelmannii* Salm = Dyck ex Engelm.	*Cactoblastis cactorum* (Berg)	Cladode sucker	Yes	Antigua South Africa	Extensive
			No	Federation of St Kitts and Nevis	-
	*Dactylopius opuntiae* (Cokerell), “*ficus-indica*” biotype	Cladode sucker	Yes	Australia South Africa	Trivial to moderate
			No	Federation of St Kitts and Nevis	-
*Opuntia ficus-indica* (L.) Mill.	*Lagocheirus funestus* (Thomson)	Stem borer	Yes	Hawaii (USA)	Considerable
				South Africa	Trivial
	*Cactoblastis cactorum* (Berg)	Cladode borer	Yes	Australia Hawaii (USA) Mauritius Puerto Rico (USA) South Africa U.S. Virgin Islands (USA)	Considerable
	*Dactylopius opuntiae* (Cokerell), “*ficus-indica*” biotype	Cladode sucker	Yes	Hawaii (USA) South Africa Spain	Considerable
				Australia	Moderate
	*Fusarium oxysporum* Schlecktendahl	Unknown	Yes	Hawaii (USA)	Unknown
	*Lagocheirus funestus* (Thompson)	Stem borer	Yes	Hawaii (USA) South Africa	Trivial
	*Melitara dentata* (Grote)	Cactus feeder	No	Hawaii (USA)	-
	*Melitara prodenialis* Walker	Cladode borer	No	Hawaii (USA)	-
	*Metamasius spinolae* (Gyllenhal)	Stem borer	Yes	South Africa	Considerable
	*Moneilema armatum* LeConte	Cladode and root borer	No	Hawaii (USA)	-
*Opuntia humifusa* (Raf.) Raf.	*Cactoblastis cactorum* (Berg)	Cladode borer	Yes	South Africa	Trivial
	*Dactylopius opuntiae* (Cokerell), “*stricta*” biotype	Cladode sucker	Yes	South Africa	Extensive
*Opuntia littoralis* (Engelm.) Cockerell	*Chelinidea tabulata* (Burmeister)	Unknown	Yes	USA	None
	*Chelinidea vittiger* Uhler	Unknown	Yes	USA	None
	*Dactylopius confusus* (Cockerell)	Cladode sucker	No	USA	-
	*Dactylopius opuntiae* (Cockerell)	Cladode sucker	Yes	USA	Extensive
	*Dactylopius tomentosus* (Lamark)	Cladode sucker	No	USA	-
	*Melitara prodenialis* Walker	Cladode borer	No	USA	-
	*Olycella junctolineella* (Hulst)	Cladode borer	No	USA	-
*Opuntia monocantha* Haw.	*Cactoblastis cactorum* (Berg)	Cladode borer	Yes	Cuba Mauritius	Considerable
				South Africa	Trivial
	*Dactylopius ceylonicus* (Green)	Cladode sucker	Yes	Australia India Madagascar Mauritius South Africa Sri Lanka Tanzania	Extensive
				Kenya	Moderate
	*Dactylopius confusus* (Cockerell)	Cladode sucker	No	Australia India South Africa	-
	*Dactylopius opuntiae* (Cockerell)	Cladode sucker	Yes	Mauritius	Considerable
*Opuntia oricola* Philbrick	*Chelinidea tabulata* (Burmeister)	Unknown	Yes	USA	None
	*Chelinidea vittiger Uhler*	Unknown	Yes	USA	None
	*Dactylopius confusus* (Cockerell)	Cladode sucker	No	USA	-
	*Dactylopius opuntiae* (Cockerell)	Cladode sucker	Yes	USA	Moderate
	*Dactylopius tomentosus* (Lamark)	Cladode sucker	No	USA	-
	*Melitara prodenialis* Walker	Cladode borer	No	USA	-
	*Olycella junctolineella* (Hulst)	Cladode borer	No	USA	-
*Opuntia robusta* J.C.Wendl. ex Pfeiff	*Cactoblastis cactorum* (Berg)	Cladode borer	Yes	Australia	Considerable
				South Africa	
	*Dactylopius opuntiae* (Cokerell), “*ficus-indica*” biotype	Cladode sucker	Yes	Australia	Considerable
				South Africa	Moderate
*Opuntia salmiana* J. Parm. ex Pfeiff.	*Cactoblastis cactorum* (Berg)	Cladode borer	Yes	South Africa	Trivial
*Opuntia spinulifera* Salm-Dyck	*Cactoblastis cactorum* (Berg)	Cladode borer	Yes	South Africa	Unknown
*Opuntia streptacantha* Lem.	*Cactoblastis cactorum* (Berg)	Cladode borer	Yes	Australia	Trivial
	*Chelinidea tabulata* (Burmeister)	Unknown	Yes	Australia	None
	*Chelinidea vittiger* Uhler	Unknown	No	Australia	-
	*Dactylopius opuntiae* (Cockerell) “*ficus-indica*” biotype	Cladode sucker	Yes	Australia	Considerable
	*Lagocheirus funestus* Thomson	Stem borer	Yes	Australia	Trivial
	*Moneilema blapsides* (Newman) subsp. *ulkei* Horn	Cladode and root borer	Yes	Australia	Trivial
*Opuntia stricta* (Haw.) Haw.	*Cactoblastis cactorum* (Berg)	Cladode borer	Yes	New Caledonia	Considerable
				Antigua Cayman Islands Cuba Federation of St Kitts and Nevis Guadeloupe Jamaica Montserrat U.S. Virgin Islands	Extensive
				Namibia South Africa	Moderate
				Australia Kenya	Trivial
				Bahamas	Unknown
	*Cactoblastis doddi* Heinrich	Cladode borer	No	Australia	-
	*Chelinidea tabulata* (Burmeister)	Unknown	Yes	Australia	None
	*Chelinidea vittiger* Uhler	Unknown	Yes	Australia	Unknown
	*Dactylopius austrinus* De Lotto	Cladode sucker	No	Federation of St Kitts and Nevis	-
	*Dactylopius ceylonicus* (Green)	Cladode sucker	No	India	-
	*Dactylopius coccus*	Cladode sucker	No	Australia	-
	*Dactylopius confusus* (Cockerell)	Cladode sucker	Yes	Australia	None
	*Dactylopius opuntiae* (Cokerell), “*stricta*” biotype	Cladode sucker	Yes	Sri Lanka	Considerable
				Australia India Kenya Saudi Arabia South Africa	Extensive
				Namibia	Moderate
				Kenya	Unknown
			No	Federation of St Kitts and Nevis	-
	*Lagocheirus funestus* Thomson	Stem borer	Yes	Australia	Trivial
	*Loxomorpha flavidissimalis* (Grote)	Cactus feeder	No	Australia	-
	*Melitara dentata* (Grote)	Cactus feeder	No	Australia	-
	*Melitara prodenialis* Walker	Cladode borer	No	Australia	-
	*Moneilema blapsides* (Newman) subsp. *ulkei* Horn	Cladode and root borer	Yes	Australia	Trivial
	*Moneilema variolare* Thomson	Cladode and root borer	Yes	Australia	None
	*Olycella junctolineella* (Hulst)	Cladode borer	Yes	Australia	None
*Opuntia tomentosa* Salm-Dyck	*Cactoblastis cactorum* (Berg)	Cladode borer	Yes	Australia	Trivial
	*Dactylopius opuntiae* (Cokerell), “*ficus-indica*” biotype	Cladode sucker	Yes	Australia	Moderate
				South Africa	Considerable
*Opuntia triacantha* (Willd.) Sweet	*Cactoblastis cactorum* (Berg)	Cladode borer	Yes	Antigua Cayman Islands Cuba Federation of St Kitts and Nevis Guadeloupe Montserrat U.S. Virgin Islands	Extensive
				Puerto Rico	Unknown
	*Dactylopius austrinus* De Lotto	Cladode sucker	No	Federation of St Kitts and Nevis	-
	*Dactylopius opuntiae* (Cokerell)	Cladode sucker	No	Federation of St Kitts and Nevis	-
*Opuntia tuna* (L.) Mill.	*Cactoblastis cactorum* (Berg)	Cladode borer	Yes	Mauritius	Extensive
	*Dactylopius ceylonicus* (Green)	Cladode sucker	No	Mauritius	-
	*Dactylopius opuntiae* (Cokerell) “*ficus-indica*” biotype	Cladode sucker	Yes	Mauritius	Trivial
*Peniocereus serpentinus* (Lag. and Rodr.) N.P. Taylor	*Hypogeococcus festerianus* (Lizer y Trelles)	Stem sucker	Yes	South Africa	Unknown
*Pereskia aculeata* Mill.	*Catorhintha schaffneri* (Brailovsky & Garcia)	Stem wilter	Yes	South Africa	Unknown
	*Phenrica guerini* Bechyné	Leaf feeder	Yes	South Africa	Moderate

## Supplementary Materials

The following are available online at https://www.mdpi.com/2223-7747/8/10/421/s1, Supplementary material: Online questionnaire.
