# Supplementary material for: Global Actions for Managing Cactus Invasions
Source: Plants (Basel). 2019 Oct 16;8(10):421. doi: 10.3390/plants8100421 (PMC6843271; doi:10.3390/plants8100421)
Supplement: Supplementary file 1 [file plants-08-00421-s001.pdf]

Supplementary material

Supplementary material 1. Online questionnaire. The results of the questionnaire are presented in red font. The numbers in brackets indicate the number of respondents for a given answer.

Thank you so much for collaborating with us by filling this questionnaire!
We really appreciate your help!

#Note: We are aware of the fact that many people know the species of cactus by its common name, and not its scientific one. However, common names differ from region to region (even within a country). Therefore, our knowledge on common names is limited. If you know the species by their common names, please, visit "http://academic.sun.ac.za/cib/projects/cactuswg/invasivecactus.asp", where you will find pictures of most invasive cactus species. Sorry for the inconvenience. Thanks!

1. **Which is your region* of expertise?**

**Region: the geographic area for which you are answering this questionnaire. Even if you are just answering about a small region, please, indicate the country where the region belongs.*

- Australia (25)
- Eastern Africa
- France (22)
- Italy (10)
- Namibia
- Portugal (10)
- South Africa (13)
- Spain (5)
- Zimbabwe
- Other: Tunisia (2), Austria (1), Kenya (2), Lesser Antilles (1), Macedonia (2), Mexico (1), Pacific Islands (1)

2. **Which is your area of expertise?**

- I am an invasive species manager (37)
- I am a property (e.g. farm) owner (8)
- I am a food scientist (2)
- I am an invasion biologist (26)
- I am a researcher on biological control (7)
- I am an horticulturist (11)
- I am a policy maker (4)
- Other: (0)

3. **Which pathways do you consider most important for the introduction of new cactus species into your region?***

- Horticulture (91)

- Human consumption and fodder (4)
- Living hedges (2)

4. Which pathways of secondary release do you consider responsible for the movement of invasive cactus species within your region?*

- Horticulture (90 respondents)
- Attached to domestic animals (80)
- Attached to wild mammals (70)
- Attached to vehicles (50)
- Disposal of garden waste (50)
- Attached to clothes (40)
- Dispersed by water bodies (20)
- Dispersed by wind (10)
- Human consumption and fodder (10)
- Living hedges (8)
- Attached to wild birds (4)
- Attached to wild reptiles (2)

5. Is the movement of alien cactus regulated within your region (i.e. cactus cannot be legally moved from one area to another within your region)?

- Yes (65)
- No (0)
- I don't know (30)

If so, could you give some details about the regulations, and clarify if the regulation is "cactus specific" or just part of "general invasive species regulations"?

- Cactus specific (0)
- General invasive species regulations (65)
    - Weeds of National Significance (WoNS) (24 respondents)
    - National Environmental Management: Biodiversity Act: List of invasive species (No. 599 of 2014). It implements the National Environmental Management Biodiversity Act, 2004 (No. 10 of 2004). 2004-05-31 "Alien and Invasive Species Regulations" (13)
    - Real Decreto Nº 630/2013 - Regula el Catálogo español de especies exóticas invasoras (5)
    - Land Protection (Pest and Stock Route Management) Act 2002 (4)
    - Biosecurity and Agricultural Management Act 2007 (3)
    - Catchment and Land Protections Act 1994 (3)
    - Natural Resources Management Act 2004 (3)
    - Regional Act No. 56 making provision for the conservation and the protection of natural and seminatural habitats, flora and wildlife and laying down amendments to Regional Act No. 7 of 23 January 1998 and to Regional Act No. 49 of 11 April 1995. (3)
    - Biosecurity Act 2015 (2)

- Plant Protection Order, 1961. Consolidated version of 2012 of L.N.744/19661 as amended last by L.N. 130/1990 (2)
- Weeds Management Act 2013 (2)
- Noxious Weeds Order (Chapter 35:04). Consolidated version of S.I. No. 49 of 1968 as at 31 December 2013 and amended by S.I. No. 84 of 1976 (1)

6. Is the introduction of new alien cactus species from other regions regulated in your region (i.e. cactus cannot be legally introduced from other regions)?

- Yes (52)
- No (13)
- I don’t know (30)

If so, could you give some details about the regulations, and clarify if the regulation is "cactus specific" or just part of "general invasive species regulations"?

- Cactus specific (0)
- General invasive species regulations (52)
  - Same as before (49)
  - Decree-Law No. 565/99 regulating the introduction of exotic flora and fauna species (3)

7. Is there any available method for cactus risk assessment in your region?

- Yes (47)
- No (2)
- I don’t know (46)

If so, could you give some details about the available methods for cactus risk assessment in your region?

- Australian Weed Risk Assessment (38)
- Risk Assessment protocol developed for central Europe by Weber and Gut in 2004 (9)

8. Is there any detection program underway to detect new cactus invasions (early warning and rapid response) in your region?, e.g. inspection teams, public app (e.g. Ispot),...

- Yes (76)
- No (0)
- I don’t know (19)

If so, could you give some details about the detection programs underway to detect new cactus invasions (early warning and rapid response) in your region?

- Citizen Science Programs (26)
  - MyWeedWatcher (2)
  - Weeds of Western Australia (2)
  - Weed Spotters Network Queensland (2)
  - invasoras.pt (2)
  - iNaturalist (5)

- Southern African Plant Invaders Atlas (6)
- Alert network to detect *Cylindropuntia Rosea* in Valencia, Spain (1)
- CyberTracker software in Kruger National Park, South Africa (1)
- Remote sensing in Kruger National Park, South Africa (1)

9. **Are you aware of any cactus species having being eradicated* from your region? If so, could you give some details about the eradication of cactus species from your region?**

**Eradication: elimination (including all seeds and vegetative propagules) of a cactus species from an area to which re-colonisation is unlikely to occur*

- Yes (0)
- No (95)

10. **To eradicate, control or contain cacti, which methods are used in your region?**

- Physical (75)
- Chemical (59)
- Biological (43)
- I don't know (3)
- Other:

11. **If you selected the option "Physical" in question 15, could you please (if possible) give more information (e.g. techniques used)?***

**If you do not have enough space, please, send the information to: novoa.perez.ana@gmail.com*

- Bulldozers (20)
- Burial (15)
- Digging hoes (10)
- Placing removed plants in water (7)
- Excavators (5)
- Shovels (5)
- Drying plants (3)
- Spades (3)
- Burning plants (2)
- Rakes (1)

12. **If you selected the option "Chemical" in question 15, could you please (if possible) specify which herbicide (or in general PPP, Plant Protection Product) is used (active ingredient, concentration and/or mixture) in your area?***

**If you do not have enough space, please, send the information to: novoa.perez.ana@gmail.com*

See table 3 in the main text for results

13. **If you selected the option "Biological" in question 15, could you please (if possible) specify which biocontrol agent is used for each species (e.g. *Dactylopius tomentosus* stricta biotype) ?***

**If you do not have enough space, please, send the information to: novoa.perez.ana@gmail.com*

See table 4 in the main text for results

14. **Is there any other method of eradication, control or containment of cactus species you would like to mention?**

Integrated management (10)

15. **Is there any education or public awareness program to promote the knowledge of cactus invasions and/or impacts underway in your region?**

- Yes (63)
- No (17)
- I don't know (15)

Please, specify*

**e.g. pamphlets, posters, newspaper publications, magazine publications, radio advertisements, TV advertisements, workshops, school visits...*

- Fact sheets (41)
- Websites (27)
- New letters (23)
- Voluntary activities (18)
- Public talks to NGOs, private or public environmental managers, school learners or universities (12)
- Books (9)
- Documentaries (4)
- Video interviews (3)

16. **Is there any public engagement program regarding cactus invasions underway in your region?**

- Yes (9)
- No (23)
- I don't know (63)

Please, specify*

**e.g. websites (e.g. invasoras.pt), volunteer programs...*

- Workshops (7)
- Volunteer programs (2)

17. **Is there anything else you would like to add to this questionnaire?**

- Lack of funding and capacity (57)
- Lack of data on the impacts of invasive cacti (53
- Taxonomic problems within the family (32)

- Lack of public awareness (7)
- Difficulties to introduce biocontrol (3)
- Lack of policy implementation (2)
- Need to engage with private owners (2)
- Lack of register herbicides (1)

Thank you!
